# Enhancing single-cell ATAC sequencing with formaldehyde fixation, cryopreservation, and multiplexing for flexible analysis

**DOI:** 10.1186/s13104-025-07547-y

**Published:** 2025-10-20

**Authors:** Tobias Hohl, Ulrike Bönisch, Thomas Manke, Laura Arrigoni

**Affiliations:** 1 Max-Planck-Institute of Immunobiology and Epigenetics, 79108, Stübeweg 51, Freiburg im Breisgau, Germany; 2 Faculty of Biology, Albert-Ludwigs-Universität Freiburg, Schänzlestraße 1, 79104 Freiburg im Breisgau, Germany; 3 School of Computing, Communication and Business, HTW Berlin, Wilhelminenhofstr. 75a, 12459 Berlin, Germany

**Keywords:** ATAC-seq, Sample preservation, Single-cell analysis, Formaldehyde fixation, Cryopreservation, Multiplexing, Batch effect

## Abstract

**Objective:**

The need for freshly isolated cells in bulk or single cell ATAC-seq experiments creates considerable logistical barriers and increases susceptibility to batch effects. This makes it difficult to coordinate complex or longitudinal studies. Our goal was to develop a sample preservation strategy that overcomes these limitations, enabling consistent and high-quality chromatin accessibility profiling from archived samples.

**Results:**

We established a workflow that incorporates mild formaldehyde fixation prior to cryopreservation, preserving both bulk and single-cell ATAC-seq data quality at levels comparable to fresh samples in HepG2 cells. This protocol reliably maintains key data quality metrics, including signal-to-noise ratio and fragment distributions. Furthermore, the method is fully compatible with transposase-based sample multiplexing using custom Tn5 barcodes. To address barcode hopping inherent to multiplexing, we introduced a computational demultiplexing strategy based on fragment ratios, which accurately assigns single cells to their sample of origin. Our approach streamlines experimental logistics and ensures reproducibility across diverse and temporally dispersed samples, broadening the scope for ATAC-seq–based studies, including those in clinical research settings where coordinated sample collection is challenging.

**Supplementary Information:**

The online version contains supplementary material available at 10.1186/s13104-025-07547-y.

## Introduction

ATAC-seq is a pivotal tool for studying chromatin accessibility, providing insights into gene regulation [1]. However, the necessity for fresh samples introduces logistical hurdles and potential technical artifacts, affecting data reproducibility and quality [2, 3]. While DMSO cryopreservation is common in ATAC-seq [4, 5], it can unpredictably alter chromatin structure and gene expression. To address these challenges, we explore a two-step procedure involving formaldehyde fixation combined with cryopreservation or flash freezing. Formaldehyde stabilizes samples, preventing biological changes during collection and storage, and extends ATAC-seq applicability to archived material. Previous studies have shown formaldehyde utility in preserving chromatin structure [6] and its effectiveness in FFPE samples [7]. Additionally, methods like FixNCut have been shown to preserve RNA integrity and minimize artifacts in single-cell assays [8].

Building on these findings, we evaluated formaldehyde fixation for single-cell ATAC-seq (scATAC-seq) using the 10x Genomics Chromium platform. By benchmarking various preservation strategies and formaldehyde concentrations, we identified optimal conditions that maintain data quality comparable to fresh samples. In particular, fixing with 0.1% formaldehyde followed by DMSO cryopreservation yields high-quality data resembling untreated samples. This approach enhances library preparation robustness and offers practical guidelines for complex designs, such as time-course experiments with multiple conditions.

Additionally, we developed a transposase-based multiplexing strategy to lower costs and improve workflow efficiency, enabling sample collection at specific time points while facilitating high-throughput analysis with the 10x Genomics platform. We anticipate this low-formaldehyde method may also benefit other Tn5-based applications, such as CUT&Tag and multiomic studies.

In summary, our study presents a comprehensive approach to overcoming fresh sample limitations in ATAC-seq, ensuring high-quality data generation while addressing cost concerns through multiplexing, paving the way for more robust and flexible study designs.

## Main text

### Results

#### Combining low formaldehyde fixation with cryopreservation yields good quality bulk ATAC-seq data

**Fig. 1:**
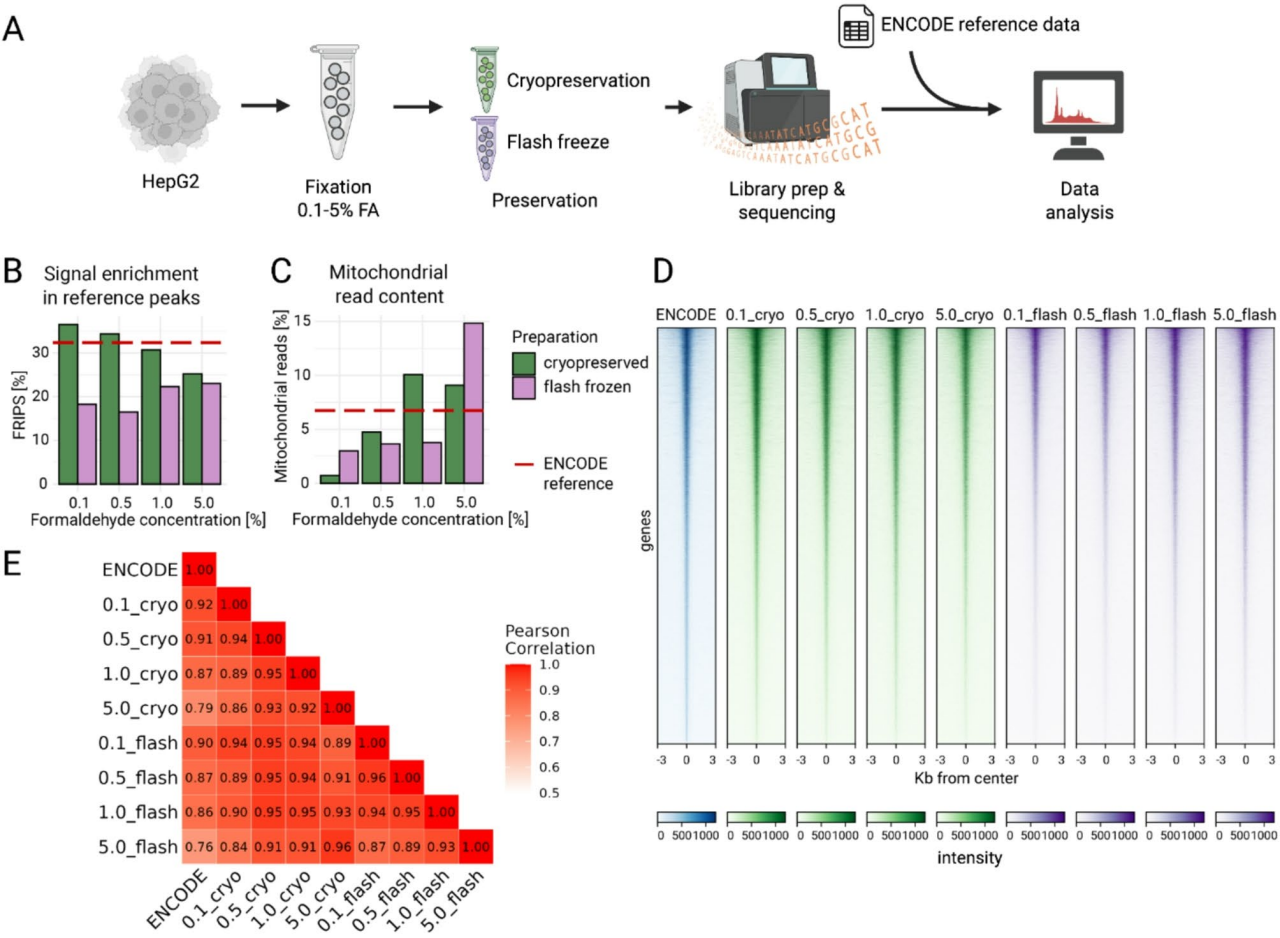
Coupling formaldehyde fixation with preservation methods yields good quality bulk ATAC-seq data. **a** Experimental workflow. HepG2 cells were formaldehyde-fixed at several concentrations, split and either cryopreserved in DMSO freezing medium (Cryopreserved) or pelletted and flash-frozen (Flash freeze). Bulk ATAC-seq libraries were prepared and sequenced. Created in BioRender. Arrigoni, L. (2024) https://BioRender.com/u59v773**b** Signal enrichment in reference peaks, measured as FRIP scores of cryopreserved and flash-frozen samples at different formaldehyde concentrations as well as the ENCODE reference data. **c**: Fraction of mitochondrial reads in sequencing data of fresh and cryopreserved samples at different formaldehyde concentrations. **d**: Heatmap showing signal enrichment centered around ENCODE reference peaks and spanning + 3 and − 3 Kb from the peak center for selected conditions. **e**: Heatmap of Pearson correlation coefficients between different conditions

HepG2 cells were treated with formaldehyde (FA) concentrations from 0.1% to 5% to analyze its effects on chromatin accessibility and assess the impact on bulk ATAC-seq data quality. While 1% FA is typical in chromatin assays, higher concentrations can enhance signal, prompting an evaluation of this effect on ATAC-seq [9, 10]. Following fixation, cells were preserved by cryopreservation or flash freezing, and ATAC-seq libraries were generated via the “Omni-ATAC” procedure [11]. Data were compared with publicly available ENCODE references, where fresh untreated cells were used (Fig. 1a).

Cryopreserved samples with low FA concentrations showed improved data quality, achieving a FRIPs score of about 35%, comparable to or even slightly higher as the ENCODE reference data, while flash frozen samples scored around 20% (Fig. 1b). Higher FA concentrations increased mitochondrial read contents and led to noisier genome track signals (Fig. 1c, Additional File 1: Figure S1), while the signal around ENCODE peak regions was comparable between all conditions (Fig. 1d). In contrast, sample-to-sample correlation (Fig. 1e, Additional File 1: Figure S2a) and PCA revealed decreased performance at 5% FA concentration, while 0.1% FA and cryopreservation showed optimal correlation with the reference in terms of fragment size distribution (Additional File 1: Figure S2b, c).

HepG2 transcription factor [12] footprint analysis indicated reduced signal strength at higher FA levels (Additional File 1: Figure S3).

Comparing the peaks from ENCODE reference data with the peaks called from the data of the cryopreserved sample fixed with 0.1% FA exhibited a substantial overlap (~ 70%), demonstrating consistency in signal while not introducing artificial peaks compared to fresh samples (Additional File 1: Figure S2d). This outcome verifies the minimal bias and reliable peak calling achievable with optimal FA usage. Furthermore, inspection of the heatmap (Additional File 1: Figure S2e) shows a very consistent signal also for those regions that were not jointly called as peaks - this reflects threshold effects and possible differences in statistical power.

Consequently, mild fixation combined with cryopreservation offers a dependable approach for maintaining high-quality ATAC-seq data, comparable to fresh samples without compromising integrity. This strategy fully validates the utilization of 0.1% FA for single-cell experiments, ensuring both data fidelity and reproducibility.

#### Evaluating preservation methods for single-cell ATAC-seq applications

**Fig. 2:**
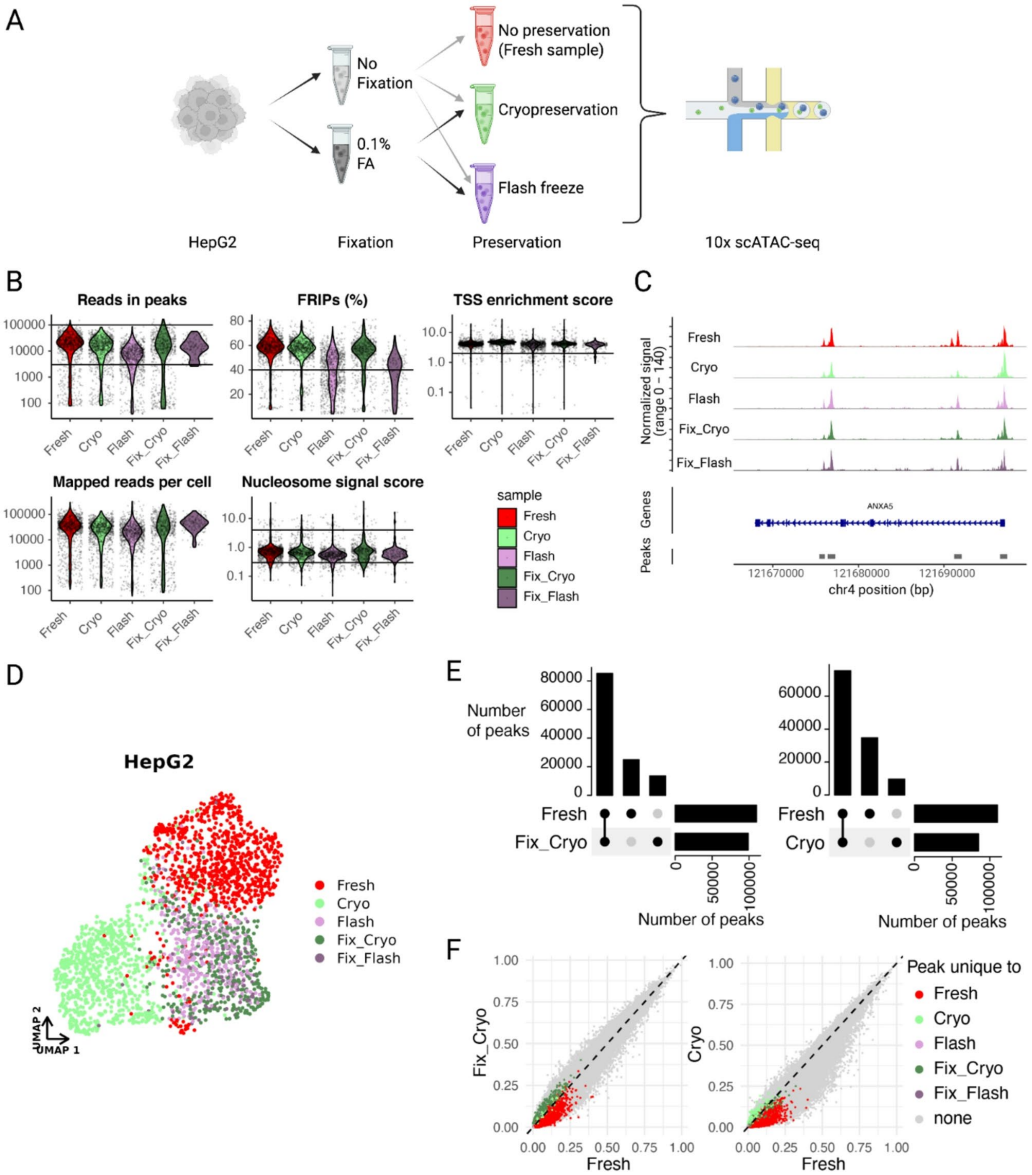
Microfluidics based scATAC-seq can be performed with fixed and preserved samples. **a** Experimental workflow. Unfixed or fixed (0.1% formaldehyde) HepG2 cells were aliquoted and preserved using cryopreservation or flash-freezing. An aliquot HepG2 not subjected to fixation and preservation (called “fresh”) was also processed in parallel. Panel created in BioRender. Arrigoni, L. (2024) https://BioRender.com/s08b492**b** Single-cell data quality metrics for each sample treatment (Fresh, Cryopreserved, Flash-frozen, Fixed and cryopreserved, Fixed and flash-frozen). The violin plots show the density of reads in peaks, FRIP scores (fraction of reads in peak regions), TSS enrichment scores, number of mapped reads per cell and nucleosome signal score. Horizontal lines depict thresholding values used for filtering during quality control. **c** Genome tracks showing the signal enrichment per sample and the identified peaks in the ANXA5 gene region. **d** UMAP representation of HepG2 scATAC-seq data. **e** Upset plots comparing peak sets called per sample. Vertical bars indicate the number of intersected peaks, or unique to the selected sample. Horizontal bars indicate the number of total peaks detected per sample. The left panel shows the peak comparison between Fresh and Fixed and cryopreserved samples. In the right panel, Fresh and cryopreserved samples are compared. **f**: Correlation plots illustrating the fractions of cells per sample that exhibit signal in the respective peak region. Each dot represents a peak among all peaks called per sample. The axes indicate the fractions of cells within the specified sample that show at least one read within the respective peak region. Peaks are colored if they were only called in one sample. The left panel compares Fresh with Fixed and cryopreserved samples. The right panel compares Fresh with cryopreserved samples

In extending our bulk ATAC-seq findings to single-cell applications, we integrated formaldehyde (FA) fixation into the 10x Genomics workflow and evaluated HepG2 cells under multiple conditions: fresh, cryopreserved, flash-frozen, 0.1% FA with cryopreservation, and 0.1% FA with flash freezing (Fig. 2a). Quality control assessments revealed comparable metrics across conditions, but flash-freezing reduced the signal-to-noise ratio captured in the FRIP score (Fig. 2b) and TSS accessibility (Additional File 1: Figure S4a) compared to cryopreservation, which produced data more comparable to fresh samples. The lower signal-to-noise ratio was also noticeable in a representative genome track (Fig. 2c). Importantly, non-fixed flash-frozen or cryopreserved samples lacked a distinct nucleosomal pattern, which was restored when adding a fixation step (Additional File 1: Figure S4b).

Differences between preservation protocols were visualized at the cellular level using UMAP embedding (Fig. 2d, Additional File 1: Figure S4c). This embedding, based on Latent Semantic Indexing (LSI) components (Additional File 1: Figure S5a), reveals protocol-specific differences. We investigated these differences further by identifying the LSI components responsible for sample separation (Additional File 1: Figure S5a). We then looked at the component loadings and investigated the top correlated regions (Additional File 1: Figure S5b-e). While these regions show increased signal in fresh samples, all sample types displayed signal in these regions (Additional File 1: Figure S5e) suggesting that the technical variation seen in the UMAP stems from protocol-specific differences in signal intensity rather than alterations of the epigenetic structure.

To further validate this point, we conducted differentially accessible regions (DARs) analysis comparing all treatments. The analysis indicated that although the fresh sample yielded the highest number of DARs (Additional File 1: Figure S6a), their characteristics were similar across all samples (Additional File 1: Figure S6b). Looking at the top DARs per condition revealed variations in signal intensity; however there was no absence of signal in samples with lower signal levels (Additional File 1: Figure S6c). This suggests that the observed DARs originate from preparation-specific signal strength variability rather than altered epigenetic profiles.

We conducted sample-specific peak calling to provide comprehensive peak sets both to compare data complexity and signal retention across samples and to perform signal presence correlation analysis (Fig. 2e and f, Additional File 1: Fig. 7). While the untreated fresh sample yielded the most peaks (~ 110.000) out of all samples (Additional File 1: Fig. 7a), the fixation step led to increased peak discovery in the cryopreserved sample (~ 100.000 vs. ~ 85.000 for cryopreserved without fixation) and gains in the number of peaks shared with the fresh sample by ~ 10.000 peaks (Fig. 2e). Preservation overall decreased the fraction of cells with reads in specific peak regions, but fixation mitigated this reduction, enhancing signal retention (Fig. 2f, Additional File 1: Figure S7b). Moreover, differentially called peaks were predominantly found in low fractions of cells (Fig. 2f), suggesting observed differences arise largely from statistical power disparities linked to varying cell numbers and sequencing depths across protocols, rather than inherent biological variance.

Our data confirm the feasibility of preservation techniques with the 10x Chromium scATAC-seq workflow; however, incorporating a fixation step pre-preservation enhances data quality, recovering signals otherwise lost. Ultimately, moderate fixation (0.1% FA) combined with cryopreservation results in data that most closely resemble untreated fresh samples.

#### Dealing with barcode hopping in tagmentation-based scATAC-seq sample multiplexing

**Fig. 3:**
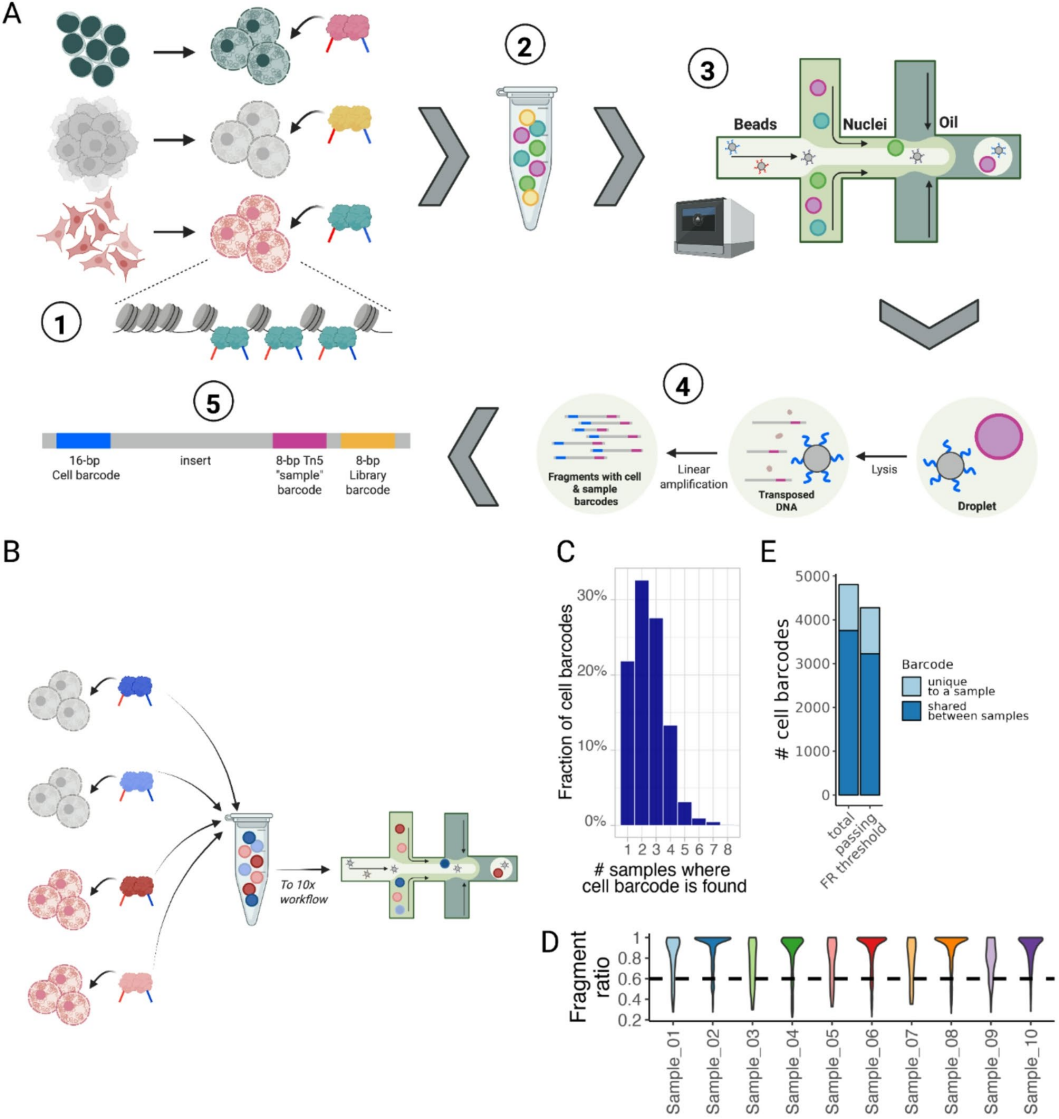
Multiplexing approach for scATAC-seq. **a** Tn5 barcoding workflow. (1) Isolated nuclei are transposed using hyperactive Tn5 with pre-loaded sample barcode constructs. (2) After Tn5 inactivation, samples are pooled. 3 & 4) 10x Genomics barcoded microbeads and transposed nuclei are encapsulated within droplets, also called GEMs (Gel beads in EMulsion). Cells are lysed inside GEMs and transposed DNA fragments are linearly amplified. At this stage single cell barcodes are incorporated in the transposed fragments. 5) PCR amplification of final fragments. The fragments include cell and library barcodes as well as the sample barcode that was introduced via Tn5 tagmentation. **b** Experimental workflow for testing the multiplexing protocol. Nuclei from individual samples were tagmented using Tn5s inserting sample-specific barcodes. After tagmentation, samples were pooled together and loaded into the 10x Genomics scATAC chip. **c** Cell barcode appearance across multiplexed samples. Only around 22% of cell barcodes appear in a singular sample, most barcodes can be found in multiple samples. **d** FR distributions across samples. The data is shown after cell barcodes were assigned to the sample with the most fragments. The dashed horizontal line depicts the FR threshold for subsequent filtering. **e**: Cell barcode retainment after FR thresholding. FR thresholding lets us recover 88% of the unique cell barcodes albeit only 22% of barcodes are unique to a sample. Panels **a**, **b** Created in BioRender. Arrigoni, L. (2024) https://BioRender.com/j56f499

Following earlier work [13], we developed a multiplexing strategy for scATAC-seq by pre-loading Tn5 enzymes with custom barcodes. After tagmentation, the samples are pooled for library preparation and sequencing following standard protocols from 10x genomics and Illumina. The data can be demultiplexed using the sample specific barcodes (Fig. 3a). We applied this workflow and pooled ten individually labeled samples for library preparation (Fig. 3b). Despite adhering to recommended loading practices, analysis revealed that only 20% of cell barcodes were unique to individual samples, with the rest appearing across multiple samples due to barcode hopping (Fig. 3c). This phenomenon aligns with observations in similar studies, where free-floating unbound Tn5 inserts led to erroneous sample barcodes and increased doublet formation, likely due to their entry into GEMs post-pooling and functioning as library preparation primers [14].

Addressing this, we introduced a bioinformatics method leveraging normalized fragment counts for robust sample demultiplexing. We assigned cellular barcodes to samples using a metric termed Fragment Ratio (FR) shown in Eq. 1, where a cell barcode is assigned to a specific sample if more than 60% of the fragments associated with a certain barcode originate from this specific sample. This metric can be calculated as follows:

1 $$\frac{{{N_{cs}}}}{{\sum\nolimits_{s} {{N_{cs}}} }}\,=\,r>0.6$$

Where:

*r* = fragment ratio.

*c* = cell barcode.

*s* = sample.

*N*_*cs*_ = fragment count seen for cellular barcode *c* in sample *s*.

FRs for all sample and cell barcode combinations are shown in Additional File 1(Figure S8a-b). For most cell barcodes, the highest contributing sample accounts for the majority of fragments, resulting in them passing the applied threshold of 60% (Fig. 3d). This refined approach considerably enhanced assignment accuracy, successfully attributing 88% of cellular barcodes to their respective samples, compared to 22% through a simple incidence model (Fig. 3e).

Despite encountering challenges with barcode hopping, our bioinformatic approach mitigates these issues, ensuring minimal data loss and precise sample assignment.

## Discussion

Disentangling the timing of sample collection from preparation is crucial for various experimental setups, where logistical constraints may prevent the timely processing of fresh samples. In our study, we demonstrate that formaldehyde fixation combined with cryopreservation preserves data integrity and produces results comparable to fresh samples, offering an effective benchmarking of preservation techniques. Fixation followed by cryopreservation particularly resembles fresh samples in signal-to-noise ratio. This method enhances reproducibility and can potentially be applied to scRNA-seq and multiome protocols. Incorporating light formaldehyde fixation before cryopreservation may help eliminate DMSO-induced artifacts on gene expression [15]. Using light rather than strong fixation could obviate the need for probe methods, as suggested by recent studies [16], thereby broadening the method’s applicability across different organisms.

Additionally, we have developed a sample multiplexing strategy that might leverage the potential of higher capacity platforms such as Chromium X, which can currently process up to 20,000 cells for scRNA-seq and may extend to scATAC-seq in the future. By collecting and fixing samples on different days, particularly those with low cell amounts, researchers can fully utilize the capacity of droplet-based methods. This approach maximizes the cost-effectiveness of high-throughput analysis tools, enabling more efficient and economical research workflows.

The multiplexing of scATAC-seq experiments has recently garnered significant attention, with various protocols emerging [17–19]. Despite challenges associated with cross-labeling due to barcode hopping, our in-silico approach using fragment ratio significantly reduces data loss, effectively addressing these concerns without additional protocol modifications.

In conclusion, we provide a multiplexed scATAC-seq workflow that allows for flexible sample collection, enabling time-course experiments to be processed within a single 10x scATAC-seq reaction. This method can mitigate batch effects arising from different sampling and library preparation experiments due to streamlined preservation and parallel processing.

## Conclusions

We present a streamlined methodology for single-cell ATAC-seq sample preparation, leveraging formaldehyde fixation to overcome fresh sample dependencies. This approach guarantees high data quality comparable to fresh samples, enabling flexible experimental designs, particularly in complex, time-course studies. Our multiplexing strategy further optimizes workflow efficiency in high-throughput platforms, providing robust solutions for sample-heavy experimental settings. While we do not yet have empirical data across different omics applications, our sample preservation strategy may offer promising solutions in future omics studies.

## Methods

All methods applied in this study are described in Additional file 3 (Extended methods).

## Limitations

This study’s findings are based solely on experiments with the HepG2 cell line, limiting generalizability. Future work should extend these methods to diverse cell types to confirm broader applicability and validate their efficacy across different biological contexts. Additionally, optimizing protocols for whole tissues remains a crucial step in enhancing the utility of our approach. The amount of formaldehyde used for fixation should be carefully assessed, particularly for more delicate cells and for mitochondria-rich cell types. Based on our findings, we advise against increasing formaldehyde concentration beyond 1% to prevent an excessive increase in mitochondrial reads.

While we propose a bioinformatic approach to deal with barcode hopping in existing multiplexing workflows, developing more refined protocols to mitigate hopping at the outset will enhance accuracy and reliability in sample assignment, ensuring robust data integrity across different experimental setups.

## Supplementary Information

Below is the link to the electronic supplementary material.

Supplementary Material 1.

Supplementary Material 2.

Supplementary Material 3.

## Data Availability

Datasets generated during this study are available in the Gene Express Omnibus repository under accession numbers GSE281808 for bulk ATAC-seq, and GSE281809 for multiplexed single-cell ATAC-seq of HepG2 cells. Bulk ATAC-Seq reference data was downloaded from ENCODE (Experiment ENCSR042AWH, library ENCLB324GIU). The reference peak set used was downloaded using ENCODE accession ENCFF314RIW. All oligonucleotide sequences are listed in Additional File 2.

## Abbreviations

ATAC: Assay for transposase-accessible chromatin
Sc: Single cell
FFPE: Formalin-fixed paraffin-embedded
GEM: Gel Beads-in-Emulsion
PC: Principal component
UMAP: Uniform manifold approximation and projection
PCA: Principal component analysis
HepG2: Hepatoblastoma cell line
QC: Quality lntrol
FRIP: Fraction of reads in peaks
ENCODE: Encyclopedia of DNA elements
TSS: Transcription start site
TF-IDF: Term Frequency - inverse document frequency
SVD: Singular value decomposition
LSI: Latent semantic indexing
RPKM: Reads per kilobase million
bam: Binary alignment and map
TF: Transcription factor
MACS2: Model-based Analysis of ChIP-seq 2
BCL: Binary base call
Bp: Base-pair
PBS: Phosphate-buffered saline
NGS: Next generation sequencing
ME: Mosaic end
HPLC: High performance liquid chromatography
DMSO: Dimethyl sulfoxide
DMEM: Dulbecco’s modified eagle medium
EMEM: Eagle’s ledium
BSA: Bovine serum albumin
EDTA: Ethylenediamine tetraacetic acid
FA: Formaldehyde
MUX: Multiplexed
DAR: Differentially accessible region
FR: Fragment ratio

## Code availability

Pipelines and scripts used to run analyses and generate figures in this study are available on github: https://github.com/TobiasHohl/scATAC-MUX-preservation.
