# Supplementary material for: Enhancing single-cell ATAC sequencing with formaldehyde fixation, cryopreservation, and multiplexing for flexible analysis
Source: BMC Res Notes. 2025 Oct 20;18:437. doi: 10.1186/s13104-025-07547-y (PMC12538879; doi:10.1186/s13104-025-07547-y)
Supplement: Supplementary file 2 — Supplementary Material 2. [file 13104_2025_7547_MOESM2_ESM.pdf]

Supplementary Information

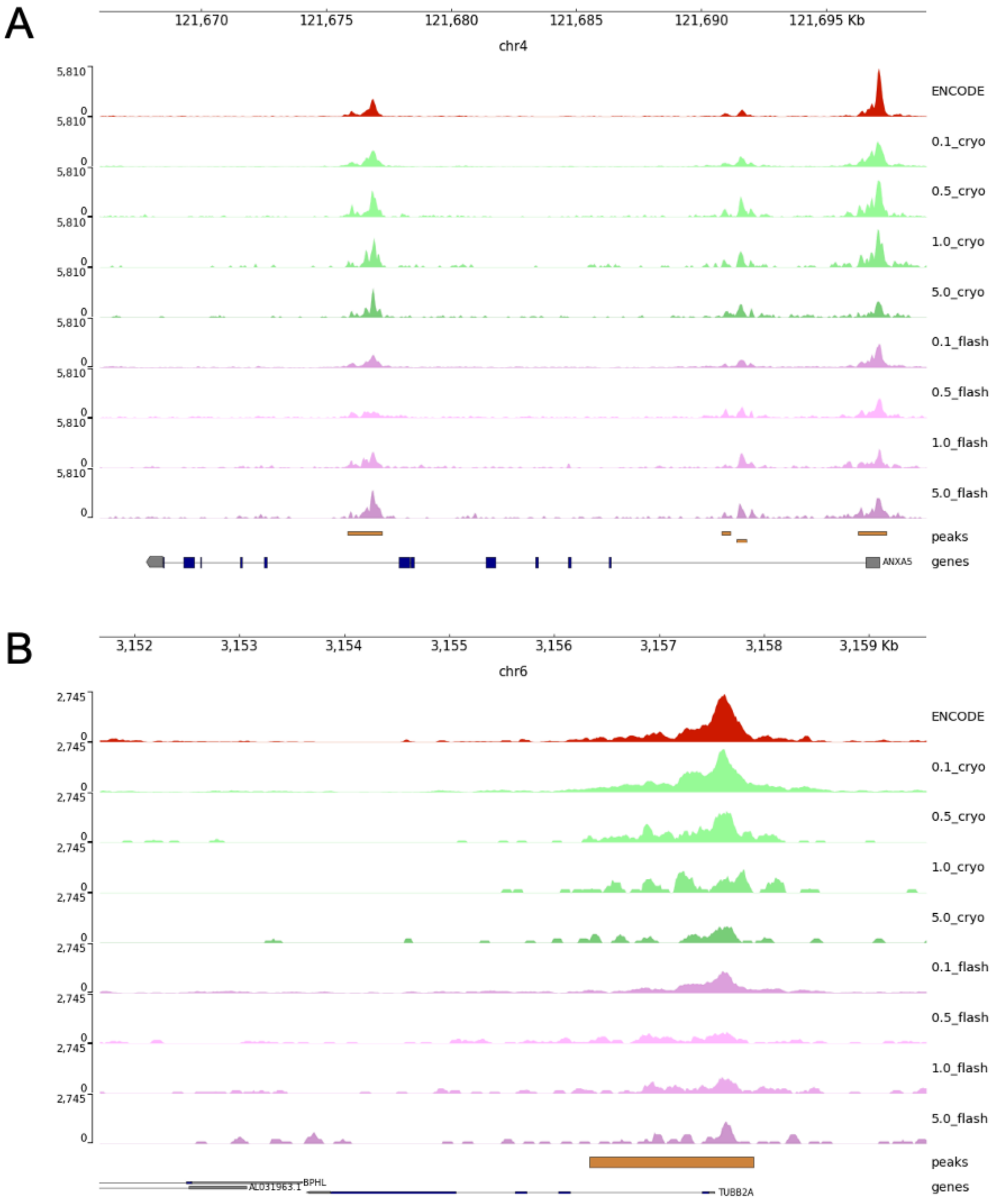

Fig. S1: Genome tracks of selected regions.

a: Genome track of the ANXA5 gene. All protocols show similar signals around peak regions. **b:** Genome track of the TUBB2A gene. While all protocols show signals within the peak region, the signal gets noisier with increasing FA concentration.

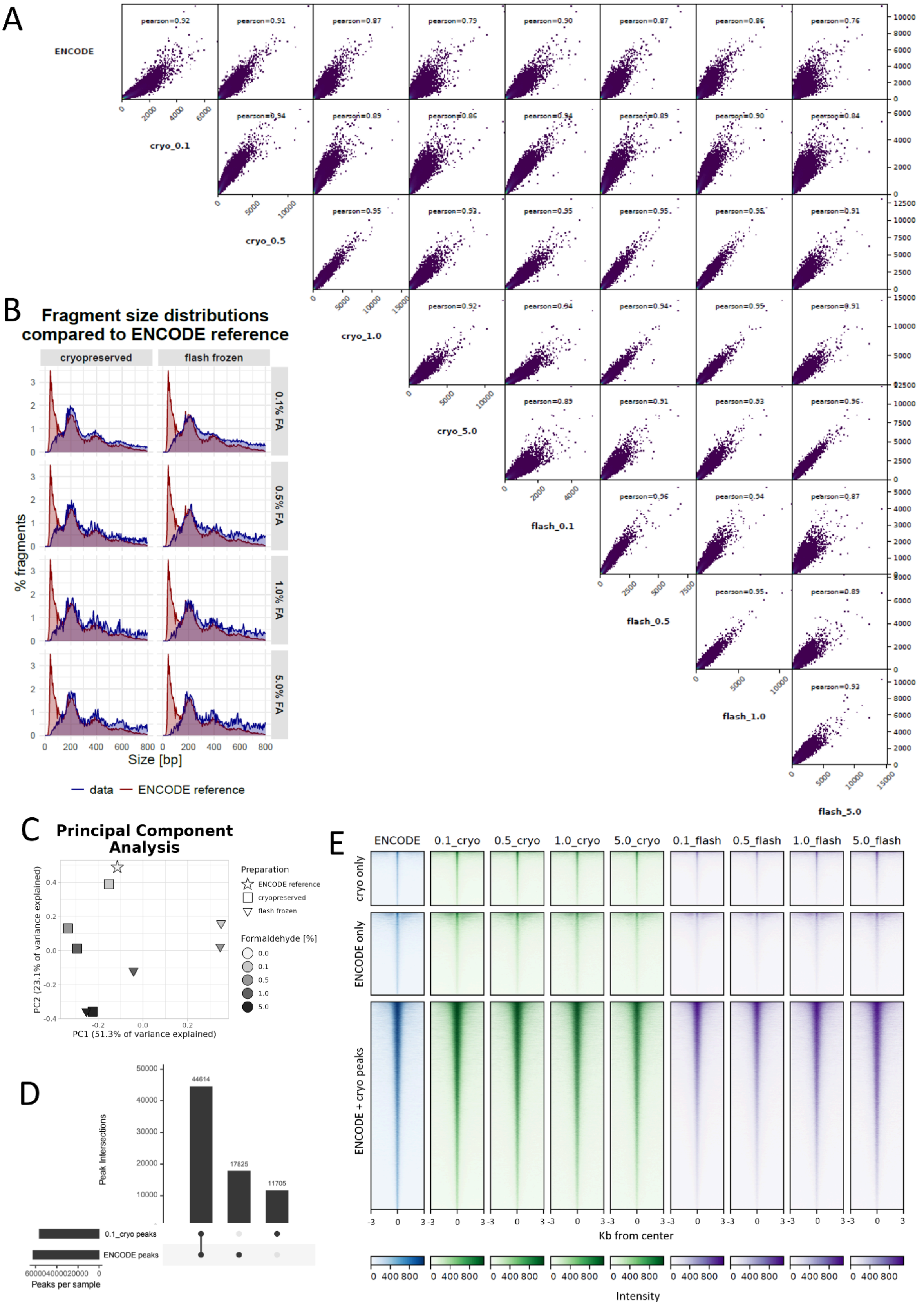
A
ENCODE
cryo_0.1
cryo_0.5
cryo_1.0
cryo_5.0
flash_0.1
flash_0.5
flash_1.0
flash_5.0
pearson=0.92
pearson=0.91
pearson=0.87
pearson=0.79
pearson=0.90
pearson=0.87
pearson=0.86
pearson=0.76
pearson=0.94
pearson=0.89
pearson=0.86
pearson=0.94
pearson=0.89
pearson=0.90
pearson=0.84
pearson=0.95
pearson=0.93
pearson=0.95
pearson=0.95
pearson=0.95
pearson=0.91
pearson=0.92
pearson=0.94
pearson=0.94
pearson=0.95
pearson=0.91
pearson=0.89
pearson=0.91
pearson=0.93
pearson=0.96
pearson=0.96
pearson=0.94
pearson=0.87
pearson=0.95
pearson=0.89
pearson=0.93
B
Fragment size distributions compared to ENCODE reference
cryopreserved
flash frozen
0.1% FA
0.5% FA
1.0% FA
5.0% FA
% fragments
Size [bp]
data
ENCODE reference
C
Principal Component Analysis
PC2 (23.1% of variance explained)
PC1 (51.3% of variance explained)
Preparation
ENCODE reference
cryopreserved
flash frozen
Formaldehyde [%]
0.0
0.1
0.5
1.0
5.0
D
Peak intersections
44614
17825
11705
0.1_cryo peaks
ENCODE peaks
Peaks per sample
E
ENCODE
0.1_cryo
0.5_cryo
1.0_cryo
5.0_cryo
0.1_flash
0.5_flash
1.0_flash
5.0_flash
cryo only
ENCODE only
ENCODE + cryo peaks
Kb from center
Intensity

Fig. S2: Further quality metrics on bulk ATAC-seq with sample preservation.

a: Correlation scatterplots showing normalized signals in reference peak regions compared between all samples. The scatterplots resemble the underlying data to the correlation analysis in Fig. 1e. **b:** Fragment size distribution for all samples compared to a reference dataset. While increasing FA concentrations yield noisier signals in the distributions, 0.1%FA cryopreserved best resembles the nucleosomal pattern found in the reference data. **c:** PCA plot of all samples. PC2 seems to capture FA concentration, and the 0.1%FA cryopreserved sample clusters closely with the ENCODE reference data. **d:** Upset plot showing the overlap between the reference peak set and a peak set called using the data from the best performing condition (0.1% FA, cryopreserved). **e:** Heatmap showing peak sets found in c. Even though peak calling was differential between the reference and the 0.1%FA cryopreserved dataset, the signal in the unique peak sets is very comparable across all samples.

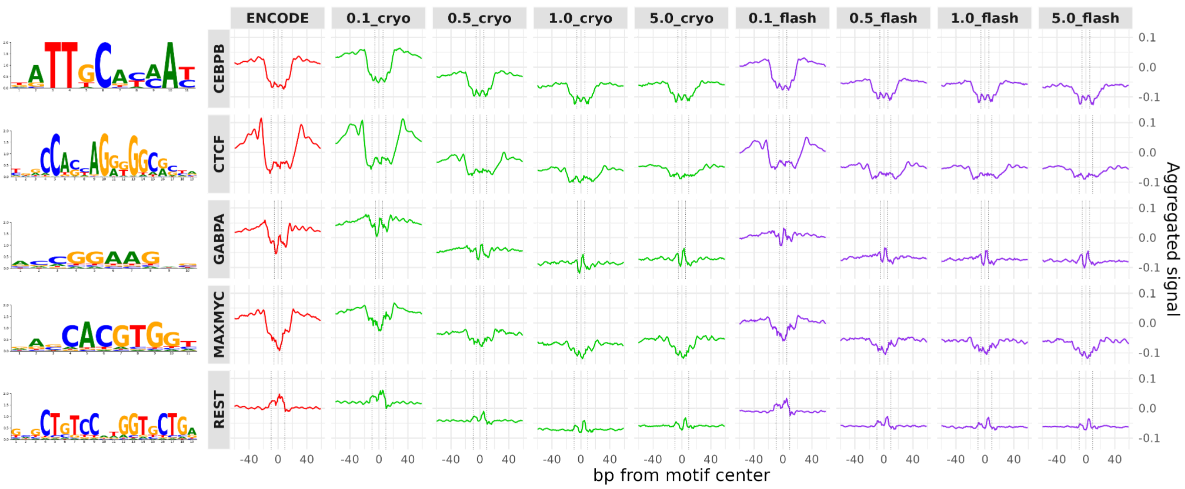

Fig. S3: Footprint plots of selected transcription factors.

Columns are samples, rows are transcription factors. Dotted lines show motif widths. While most footprints can be recovered for each sample, the strength decreases with increasing formaldehyde concentration. The GABPA footprint is visible only within the ENCODE and cryo_0.1 samples.

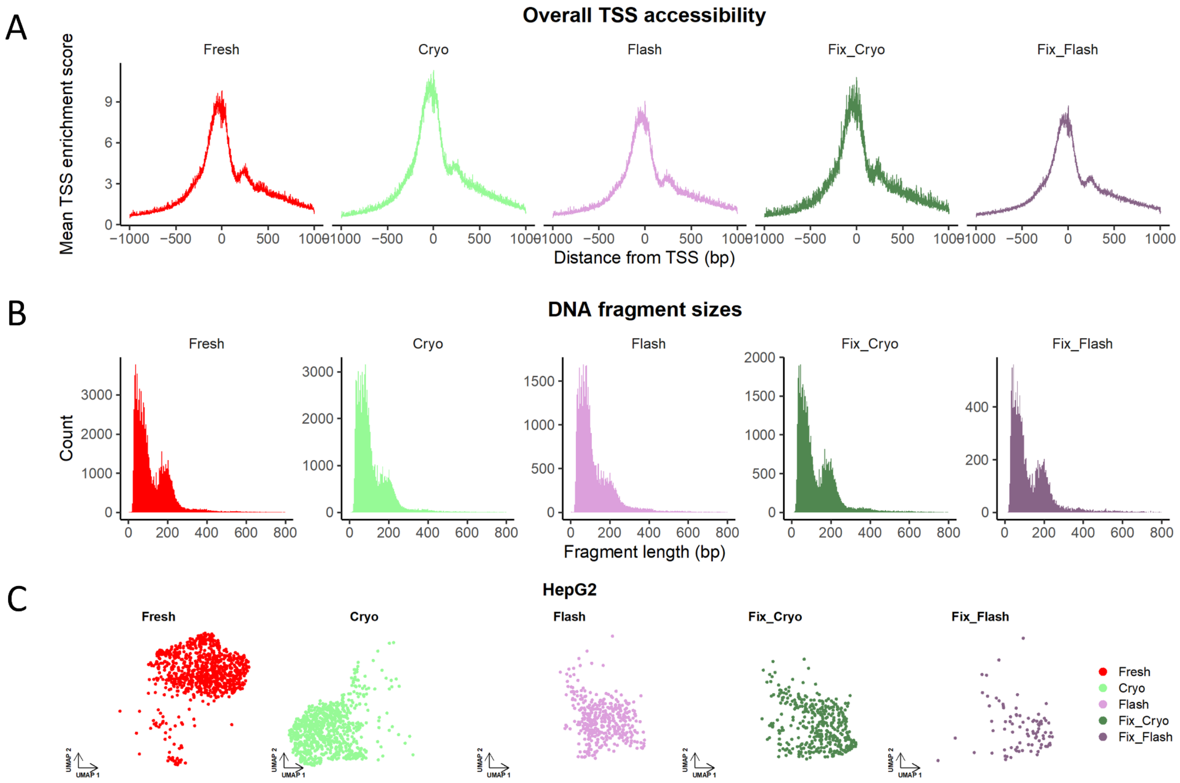

Fig. S4: Quality control of HepG2 scATAC-seq data.

a: Mean TSS enrichment score around TSS per sample. Flash freezing seems to lower the peak of the distributions. **b:** Per sample Fragment size histograms. The bimodal pattern of sub- and mononucleosomal fragments seen in the Fresh sample gets lost when cryopreserving or flash freezing. Fixation before freezing rescues the pattern. **c:** UMAP embedding split by sample.

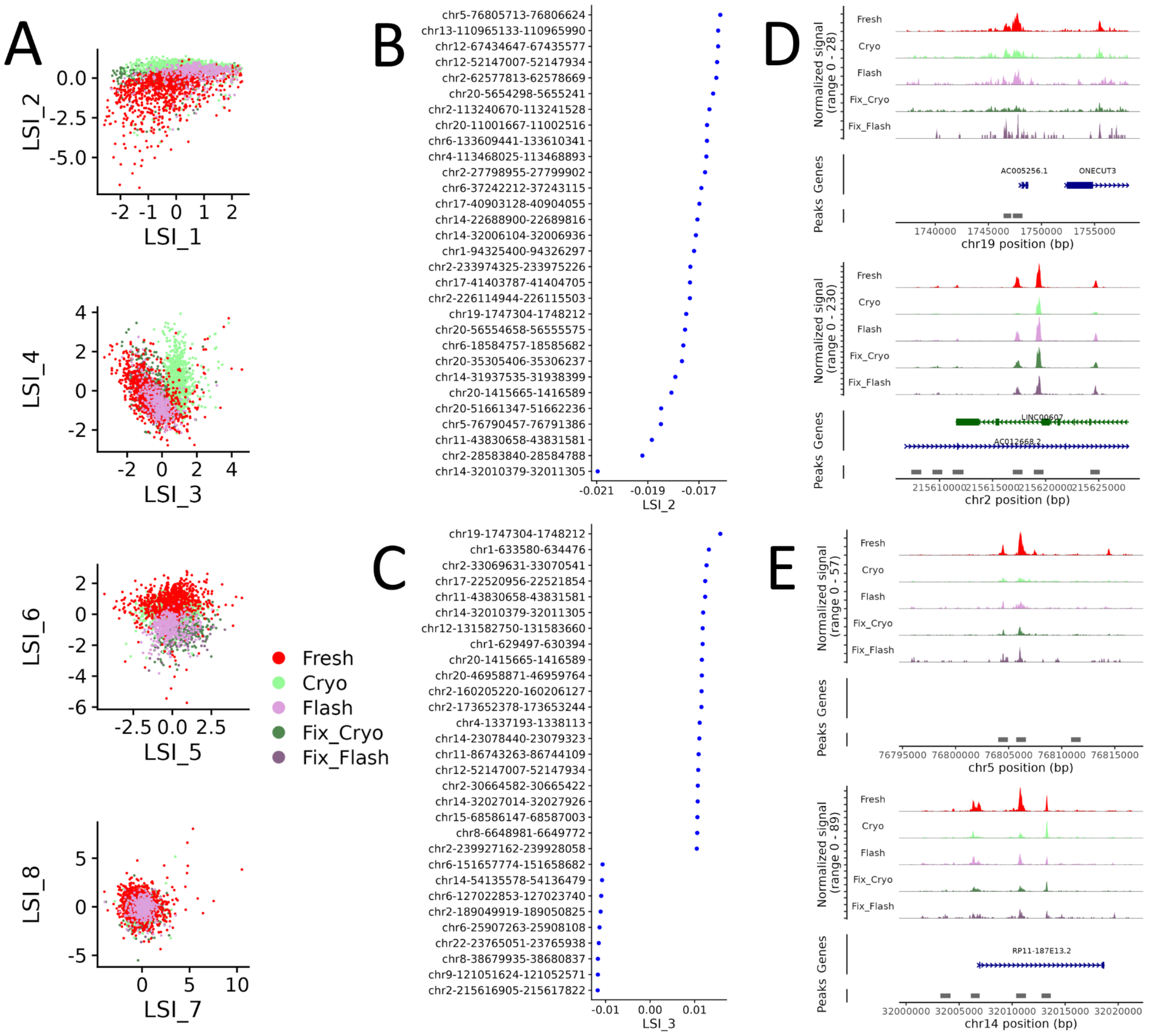

Fig. S5: Investigating the LSI-based separation of samples on the UMAP embedding.
a: Scatterplots of several LSI components colored by sample. LSI 2 and 3 show the highest degrees of sample separation. **b,c:** Loadings of LSI components 2 and 3 visualizing the top associated regions. **d,e:** Genome tracks of the top associated regions of LSI components 2 and 3. Most regions are covered by signal in all samples while the signal in the Fresh sample is increased compared to the other samples.

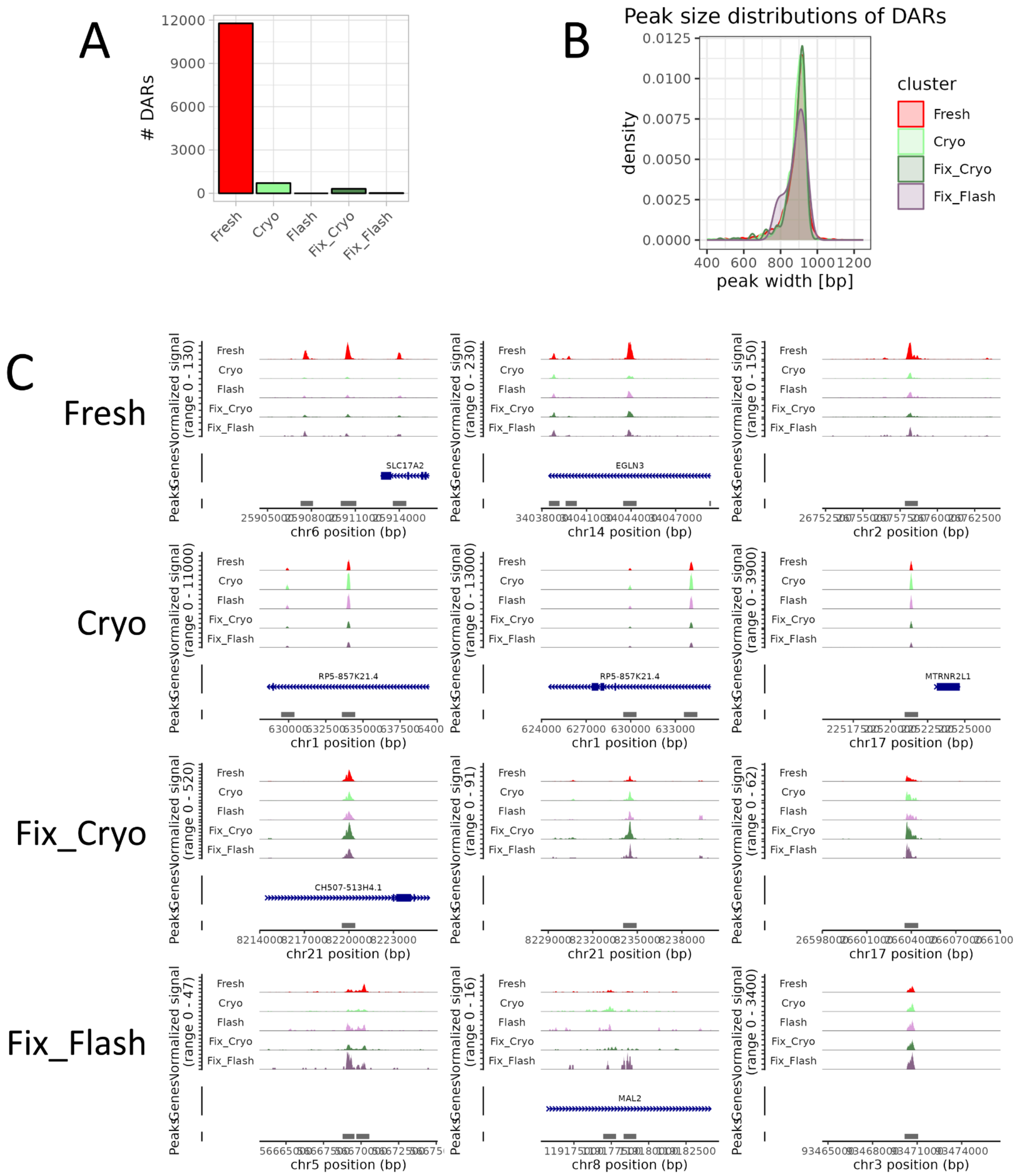

Fig. S6: Differentially accessible peaks between conditions in scATAC-seq data.
a: Amount of DARs per sample. Here, samples were compared to all other samples in order to determine DARs. **b:** Peak size distributions of DARs. All samples show very similar behavior of the distributions. **c:** Genome tracks of top 3 DARs per sample. The Flash sample did not yield any DARs. While each sample exhibits higher signal in the respective sample-specific DARs, there is signal present in all samples.

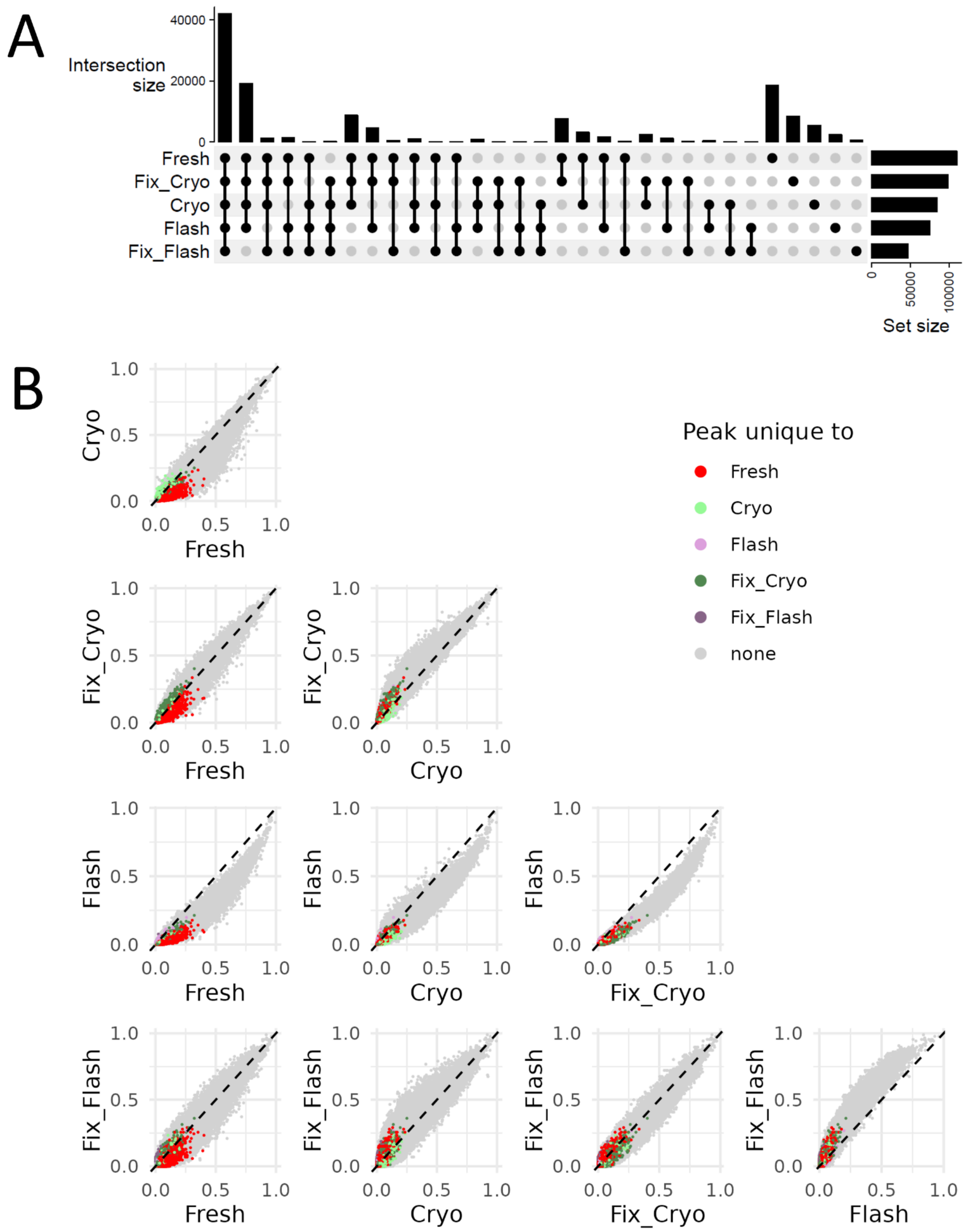

Fig. S7: Sample specific peak calling.

a: Upset plot of called peaks over all samples. Only a small percentage of peaks are unique to a certain sample. **b:** Correlation plots of peak appearance between samples. Each dot represents a peak of all peaks called per sample. The axes represent the fractions of cells within a certain sample that show at least one read within the respective peak region. Peaks are colored if they were only called in one sample. Curved plots show signal loss in certain samples, mostly non-fixed samples, while fixation prior to any freezing method seems to rescue the signal loss.

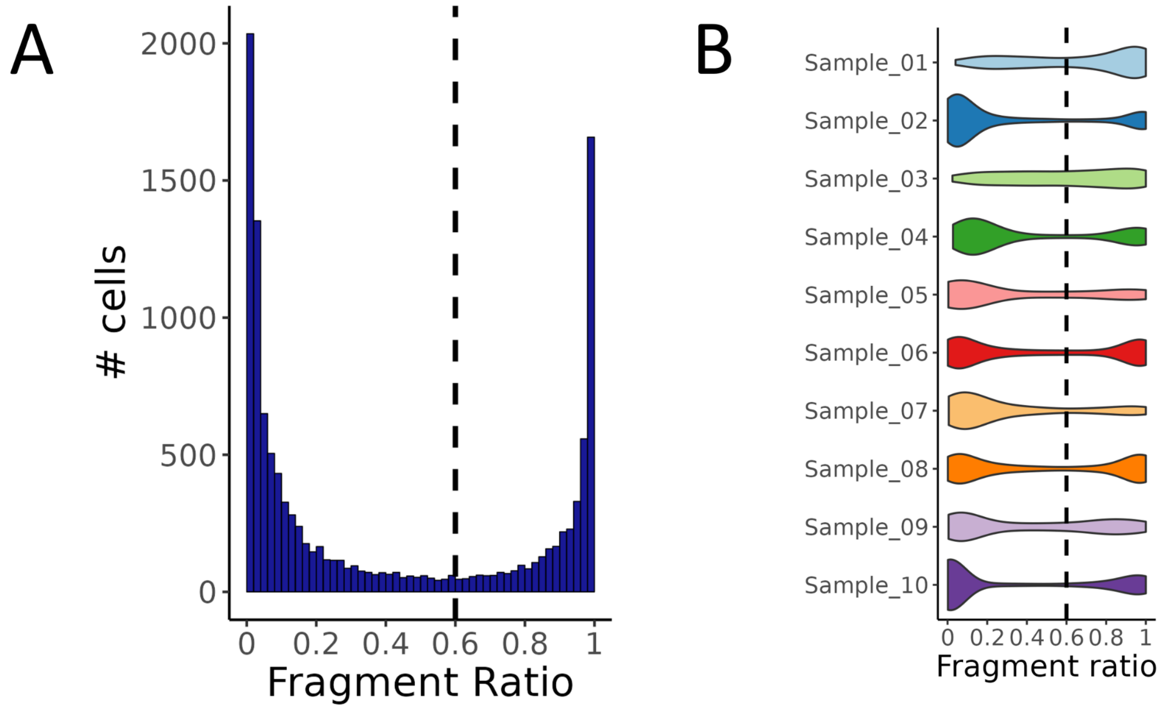

Fig. S8: Sample barcode hopping in the context of MUX-scATAC-seq.

a: Histogram of fragment ratios for all sample and cell barcode combinations. Most sample and cell barcode combinations show rather high or rather low fragment ratios. **b:** Violin plot of fragment ratios per sample for all sample and cell barcode combinations. We see a large fraction of cell and sample barcode combinations that exhibit very low fragment ratios, as already seen in panel **a**.
