# Supplementary material for: Enhancing single-cell ATAC sequencing with formaldehyde fixation, cryopreservation, and multiplexing for flexible analysis
Source: BMC Res Notes. 2025 Oct 20;18:437. doi: 10.1186/s13104-025-07547-y (PMC12538879; doi:10.1186/s13104-025-07547-y)
Supplement: Supplementary file 3 — Supplementary Material 3. [file 13104_2025_7547_MOESM3_ESM.pdf]

Extended Methods

Cell culture

Human HepG2 cells (ATCC, HB-8065) were cultured in EMEM (Sigma-Aldrich M5650) supplemented with 10% Fetal Bovine Serum (Sigma-Aldrich, F7524), 2 mM GlutaMAX (Gibco, 35050038), and 1mM sodium pyruvate (Gibco, 11360070). Cells were cultured in an incubator at 37°C with 5% CO_2. Cell passages were performed by trypsinization.

Fixation, cryopreservation and thawing

Fixative buffer was prepared by diluting a freshly opened vial of 16% Formaldehyde (CST, 12606) to a concentration of 0.1% to 5% in serum-free DMEM (Gibco, 10569010). Up to 3 million cells in suspension were transferred into 1.5 ml tubes and pelleted at 300 × g for 5 minutes. After removing the media, pellets were resuspended in 1 ml of fixative buffer and incubated at room temperature for 5 minutes, with tubes inverted every 1-2 minutes to prevent cell sedimentation. The glycine step was omitted, and cells were directly pelleted at 300 × g for 5 minutes. The supernatant was discarded appropriately. Fixed cells were washed in 1 ml of DMEM supplemented with 0.1% BSA, pelleted again, and either snap-frozen by submerging the tubes in liquid nitrogen or cryopreserved. Both snap-frozen and cryopreserved cells were stored at -70°C for long-term preservation.

Fixed or fresh cell pellets containing up to 3 million cells were resuspended in 1 ml of room temperature cryopreservation buffer (10% DMSO, 90% Fetal Bovine Serum). The vials were quickly transferred into a Mr. Frosty container equilibrated at room temperature and then placed in a -70°C freezer for at least overnight storage. For thawing, cryopreserved cells were quickly defrosted for up to 2 minutes in a thermoblock at 37°C, removing the vials when only a small ice crystal remained. Cell suspensions in DMSO were mixed and added dropwise to a 5 ml tube containing 4 ml of 1% BSA in PBS. Tubes were mixed by inversion and centrifuged at 300 × g for 5 minutes. Cells were then resuspended in the desired volume of fresh PBS/BSA and placed on ice.

Nuclei isolation

Cells in suspension were pelleted at 300 × g for 5 minutes, resuspended in fresh media, and placed on ice. Using a hemocytometer, cells were counted, and up to 2 million cells were transferred into 1.5 ml tubes and pelleted at 300 × g for 5 minutes at 4°C. Cells and nuclei were permeabilized in 100 µl of lysis buffer (10 mM Tris-HCl, pH 7.4, 10 mM NaCl, 3 mM MgCl2, 0.1% Igepal CA-630, 0.1% Tween-20, 0.01% Digitonin, 1% BSA) and incubated for 5

minutes on ice. One milliliter of ice-cold wash buffer (10 mM Tris-HCl, pH 7.4, 10 mM NaCl, 3 mM MgCl2, 0.1% Tween-20, 1% BSA) was added to the cell suspension and mixed by inversion.

For bulk ATAC experiments, an aliquot containing 50,000 nuclei was separated into a 1.5 ml tube, centrifuged at 500 × g for 5 minutes at 4°C, and resuspended in the transposition mix as detailed in the protocols below.

For single-cell ATAC protocols, nuclei in wash buffer were centrifuged at 500 × g for 5 minutes at 4°C. Supernatants were immediately discarded, and pellets were resuspended in 1x Nuclei Dilution Buffer (10x Genomics, 2000207) to achieve a nuclei concentration of 3,200 nuclei/μl.

Tn5 Barcode design and transposome assembly

Barcode design: We developed a method to determine suitable sample barcodes that could be used for scATAC-seq multiplexing (see github repository). Given the parameters barcode length, minimum and maximum GC content, minimum distance between barcodes, the method to determine sequence distances (Hamming or Levenshtein), and an optional predefined barcode, all possible barcodes are generated and subsequently filtered for GC content, no base triplication, only single base duplication, and representation of all bases. If no barcode is predefined, a barcode with a distance to its reverse complement passing the threshold is randomly selected from the filtered list. Otherwise, the predefined barcode is used as the starting point. The remaining barcode list is filtered for elements and their reverse complement passing the distance threshold to the starting barcode. Then this step is repeated - a new barcode is sampled from the remaining list, and the list is filtered using distances of barcodes and their reverse complements to the sampled barcode - until no barcodes are remaining. The result is a list of suitable sample barcodes with a defined minimum distance between each other.

All Tn5 8-nucleotide barcodes used for this study were designed to maximize sequence diversity while ensuring a 50% GC content.

Transposase loading: Oligonucleotides were purchased from IDT, with HPLC purification selected for Mosaic End adapters A and B (ME-A and ME-B), and standard desalting for the Mosaic End reverse (ME-rev). A comprehensive list of all barcodes located in the ME-B oligos is provided in Additional File 2. Lyophilized ME-A, ME-B and ME-rev oligos were resuspended in Annealing Buffer (40mM Tris-HCl pH 8.0, 50mM NaCl) to a stock concentration of 100 μM. ME-A and ME-B oligos were annealed to the complementary common ME-rev oligo. For annealing, ME-A (or ME-B) oligo was mixed with ME-rev at 1:1 ratio and incubated in a thermocycler at 95 ˚C for 5 minutes. The mixture was then cooled to

65°C at a rate of -1°C/second, incubated at 65°C for 5 minutes, and further cooled to 4°C at the same rate.

To assemble the transposome, 5 µl of each annealed A and B transposome were combined with 10 µl of unloaded Tagmentase (Diagenode, C01070010), briefly vortexed, and incubated at 23°C for 30 minutes in a thermocycler. Subsequently, 10 µl of glycerol were added to the assembled transposase for storage at -20°C. The optimal dilution ratio of the stock Tn5 should be determined experimentally.

Custom transposase titration using bulk ATAC-seq

To determine the optimal concentration of custom Tn5, libraries were prepared following the Omni ATAC-seq protocol with minor modifications and compared to those obtained using Illumina or 10x Genomics Tn5.

Nuclei suspension aliquots containing 50,000 nuclei were centrifuged at 500 × g for 5 minutes at 4°C. Each pellet was then resuspended in 47.5 µl of transposition mix (comprising 25 µl of Diagenode 2x Tn5 reaction buffer, 0.5 µl of 1% Digitonin, 0.5 µl of 10% Tween-20, 16.5 µl of PBS, and 5 µl of water) and placed on ice. Two-fold serial dilutions of the annealed Tn5 were prepared by diluting the stock Tn5 in Transposase dilution buffer (Diagenode, C01070011). To initiate transposition, 2.5 µl of custom diluted Tn5 (1:2 and 1:4), undiluted stock, ATAC enzyme B (10x Genomics, 2000265/72), or Illumina TDE1 Tagment enzyme (20034197) were added to each reaction, mixed by pipetting, and incubated for 30 minutes at 37°C in a thermocycler.

DNA was purified using the MinElute PCR purification kit (Qiagen, 28004) according to the manufacturer's instructions, with a final elution volume of 21 µl. For PCR amplification, 10 µl of each transposed DNA was mixed with 2.5 µl of 25 µM i5 primer (same primers as dual index Cut&Tag libraries (20), see also Additional File 2), 2.5 µl of 25 µM i7 primer (for 10x Genomics/Illumina Tn5, the Buenrostro 2015 list was used (20); for custom Tn5, see Additional File 2), and 25 µl of 2x NEBNext Ultra II Q5 Master Mix (NEB, M0544). The mixture was amplified (72°C for 5 min, 98°C for 30 s, followed by 10 cycles of 98°C for 10 s, 63°C for 30 s, 72°C for 1 min, hold at 4°C).

Final libraries were cleaned using Ampure XP at a 0.8x ratio (40 µl of beads per 50 µl of reaction) and eluted in 25 µl of EB (Qiagen). Libraries were quantified using the Qubit High Sensitivity DNA Assay (Invitrogen, Q32851) and inspected for size distribution using a Fragment Analyzer with the NGS 1-6000 bp hs DNA kit. The optimal concentration of custom Tn5 for the single-cell assay was determined by ensuring the closest similarity in library size distribution to those obtained using Illumina or 10x Genomics Tn5.

Multiplexed bulk ATAC sequencing

A pellet containing 50,000 nuclei was resuspended in 50 µl of transposition mix, which included 25 µl of Diagenode 2x Tn5 reaction buffer, 0.5 µl of 1% Digitonin, 0.5 µl of 10% Tween-20, 16.5 µl of PBS, 5 µl of water, and 2.5 µl of Custom Tn5 at a 1:4 dilution. Barcoded Tn5 were selected to ensure no barcode overlap after pooling. The mixture was thoroughly mixed by pipetting and incubated for 30 minutes at 37°C in a thermocycler, with the lid set at 50°C.

Transposition was quenched by adding 50 µl of 2x Tn5 stop solution (40 mM EDTA, 2 mM spermidine) to each reaction, followed by incubation at 37°C for 15 minutes in a thermocycler. The reactions were then cooled and pooled in a 1.5 ml tube on ice. The pooled cells were centrifuged at 500 × g for 5 minutes at 4°C and resuspended in 100 µl of buffer EB (Qiagen). For fixed samples, prior to purification, samples were de-crosslinked at 68°C for 30 minutes in a thermocycler, with the lid set at 85°C.

DNA was purified using the MinElute PCR purification kit (Qiagen, 28004) according to the manufacturer's instructions, with a final elution volume of 21 µl. For PCR amplification, 10 µl of each transposed DNA was mixed with 2.5 µl of 25 µM i5 primer (same primers as dual index Cut&Tag libraries (20), see also Additional File 2), 2.5 µl of 25 µM i7 primer (MUXscATAC_i7_n primer, see Additional File 2), and 25 µl of 2x NEBNext Ultra II Q5 Master Mix (NEB, M0544). The mixture was amplified (72°C for 5 min, 98°C for 30 s, followed by 10 cycles of 98°C for 10 s, 63°C for 30 s, 72°C for 1 min, hold at 4°C).

Final libraries were cleaned using Ampure XP at a 0.8x ratio (40 µl of beads per 50 µl of reaction) and eluted in 25 µl of EB (Qiagen).

10x Genomics single-cell ATAC v2 workflow

Five microliters of permeabilized nuclei suspension, at a concentration of 3,200 nuclei/µl, were mixed with 7 µl of ATAC Buffer B (10x Genomics, 2000193) and 3 µl of ATAC Enzyme B (10x Genomics, 2000265/72). The transposition reaction was incubated in a thermocycler at 37°C for 30 minutes, with the lid set at 50°C. The single-cell ATAC v2 protocol was then continued from step 2.1 of the manufacturer’s manual (Chromium Next GEM Single Cell ATAC Reagent Kits v2 User Guide, CG000496 rev B). Final libraries were PCR-amplified using 7 cycles, as recommended for a target recovery of 10,000 cells.

Multiplexed single-cell ATAC sequencing

A 5 µl aliquot of permeabilized nuclei suspension, at a concentration of 3,200 nuclei/µl, was added to 8.5 µl of transposition mix for multiplexed scATAC (comprising 7.5 µl of 2x

Diagenode Tn5 reaction buffer and 1.5 µl of 10x Genomics 1x Nuclei Dilution Buffer) on ice. To each transposition reaction, 1 µl of custom barcoded Tn5 at a 1:4 dilution was added, ensuring the selection of Tn5 barcodes to avoid overlap after pooling. All reactions were mixed by pipetting and incubated in a thermocycler at 37°C for 30 minutes, with the lid set at 50°C.

Transposition was quenched by adding 1 µl of 300 mM EDTA to each reaction, followed by incubation at 37°C for 15 minutes in a thermocycler. The reactions were then cooled and pooled in a 1.5 ml tube on ice. The pooled nuclei were centrifuged at 500 × g for 5 minutes at 4°C and washed once with 500 µl of 1x Nuclei Dilution Buffer (10x Genomics, 2000207). Transposed cells were re-counted and diluted using 1x Nuclei Dilution Buffer to a concentration of 1,040 nuclei/µl.

Fifteen microliters of the diluted cell suspension were aliquoted into a PCR tube strip on ice for loading into the Chromium Controller, with a total of 15,600 cells loaded according to 10x Genomics recommendations. The single-cell ATAC v2 protocol was then continued from step 2.1 of the manufacturer's manual (Chromium Next GEM Single Cell ATAC Reagent Kits v2 User Guide, CG000496 rev B).

For final PCR amplification at step 4.1 of the User Guide, the "individual single index set N" was replaced with 2.5 µl of 25 µM custom oligo (MUXscATAC_i7_n, sequences in Additional File 2), selecting library indices appropriately to prevent equal index assignment. Library amplification was performed using 9 PCR cycles.

Library quality assessment and sequencing

Libraries were quantified using the Qubit High Sensitivity DNA Assay (Invitrogen, Q32851), and size distribution was visualized by capillary electrophoresis using the Fragment Analyzer with the NGS 1-6000 bp High Sensitivity DNA kit. DNA concentrations were adjusted based on the percentage of library fragments between 150 to 1200 bp, as determined by smear analysis. Library molarity was calculated using the adjusted DNA concentration and the average fragment size from the smear analysis.

Libraries were pooled, cleaned of adapter dimers, denatured according to Illumina guidelines, and sequenced paired-end with a read length of 16x50x50x32 bp (i5 index, R1, R2, i7 index; see Additional File 3 for 32-bp i7 index sequences) on a NovaSeq 6000 instrument. This setup was used for sequencing the single-cell and Tn5 sample barcodes along with the insert. In cases where only the Tn5 inserted barcode is desired to be read in i7, libraries can be sequenced with a read length of 16x50x50x32 bp. The 8-bp and 32-bp index list is provided in Additional File 2.

BCL conversion and demultiplexing

BCL files were converted to fastq format using bcl2fastq2. Demultiplexing was performed using either Illumina p7 barcodes or sample barcodes introduced via multiplexing.

Bulk ATAC-seq data analysis

Trimming & mapping

Demultiplexed fastq files from our experiments as well as the reference data downloaded from ENCODE were used to run the DNA-mapping pipeline from snakePipes (v2.7.2) (21) with default parameters (for reference, refer to ourgithub repository). Briefly, ATAC cut sites were trimmed from uniquely mapped pairs (mapq > 2) using cutadapt with parameters "--trimmerOptions -a nexteraF=CTGTCTCTTATA -A nexteraR=CTGTCTCTTATA" (added in DNA-mapping call). Mapping was performed on the human genome version GRCh38 using Bowtie2 and PCR duplicates were removed using samtools.

Preprocessing of the ENCODE reference peak set

The reference peak set was downloaded, decompressed using gunzip and sorted via 'sort -k1,1 -k2,2n'. In order to get rid of duplicate peaks, the 'bedtools merge' command from the BEDTools suite was used.

Further analyses

The output of the DNA-mapping pipeline was used for further analysis. For all peak-dependent analyses, the preprocessed peak set of the reference ENCODE data was used. Most of the processing was conducted using the deepTools suite (v3.5.5) (22). Parameters for each step can be found on the github repository. Briefly, Coverage tracks were created using bamCoverage with RPKM normalization and a bin size of 1bp. Fragment size distribution data was generated using bamPEFragmentSize. FRIPs was determined using plotEnrichment. Both PCA (via plotPCA) and correlation (via plotCorrelation) analyses were performed using the output of MultiBigWigSummary. Peak coverage heatmaps were generated using computeMatrix and plotHeatmap. Genome tracks were plotted using pyGenomeTracks (23) with the generated bigwig files. The respective configuration files can be found on the github repository. Peaks for the 0.1% FA cryo sample were called using MACS2 with standard parameters. Bedtools2 was used for overlapping with the reference peak set, and the results were visualized using the UpSetR library. The TOBIAS package was used for TF motif footprinting analysis (24). Plots were generated using R (v4.3.3) and

the tidyverse, ggbeeswarm, and ggstar packages. The respective scripts can be found in the repository.

scATAC-seq data analysis

Preprocessing

Demultiplexed fastq files were processed by running the count pipeline of the CellRanger-ATAC software (v2.1.0) by 10x Genomics on each individual sample. Briefly, the pipeline filters all reads and aligns them to the reference genome, in our case GRCh38. It demultiplexes cellular information using the cell barcode, identifies transposase cut sites, calls accessible peaks across the sample, and calls cells by determining high quality cell barcodes rich in signal-specific fragments.

The peak set used for downstream analysis was generated by running CellRanger-ATAC (v2.1.0) on publicly available HepG2 scATAC-seq data (ENCODE experiment ENCSR398OHC, R1 ENCFF074MZG, R2 ENCFF971PQX, cell barcode ENCFF084OJB).

Analysis

The fragment files and cell calling information provided by the results of the cellranger-ATAC count pipeline were used to input the single-cell information to the R-based Signac framework (v1.13) (25) that served for most of the downstream analyses (see github repository). Briefly, count matrices were produced using the previously generated reference peakset and the sample-specific fragment files and cell calling information. After merging the count matrices, nucleosome signal and TSS enrichment scores are calculated. QC is performed by evaluating and filtering for FRIPS, reads in peaks, TSS enrichment and nucleosome signal scores. Subsequently, dimension reduction is performed by employing TF-IDF and SVD to generate LSI components. These serve as the basis for non-linear dimensionality reduction using UMAP. Differentially accessible regions were called using Signac’s FindAllMarkers function with a log fold change threshold of 0.25 and a minimum fraction of cells containing the respective DARS of 5%. DARs were subsequently filtered for adjusted p-values of <0.1. Sample specific peaks were called using CallPeaks after accounting for sequencing depth differences that could significantly affect peak calling by subsampling to similar cell numbers. Resulting peak sets were compared using bedtoolsr and the UpSet function from the ComplexHeatmaps package. The resulting peak sets were combined and used to create a count matrix for all samples. This count matrix was binarized and then used to determine the fraction of cells per sample that show signal in each respective region / peak.

Analysis of multiplexed scATAC-seq data

The data was preprocessed using the cellranger-ATAC count pipeline and read into the Signac framework, as previously described. To determine cross contamination of sample specific multiplexing barcodes, the occurrence of cell barcodes across samples was determined. We then developed a method to bioinformatically allocate unique cell barcodes to the respective samples. We make cell-sample assignments based on the normalized fragment counts seen for cellular barcode c in sample s. Briefly, we will assign a cellular barcode c to sample s if more than 60% of all fragments with cell barcode c derive from sample s (see Formula (1)). Downstream analysis followed the pipeline described above.
